# Supplementary material for: Whole-Genome Cartography of Estrogen Receptor α Binding Sites
Source: PLoS Genet. 2007 Jun 1;3(6):e87. doi: 10.1371/journal.pgen.0030087 (PMC1885282; doi:10.1371/journal.pgen.0030087)
Supplement: Supplementary file 1 [file pgen.0030087.sg001.pdf]

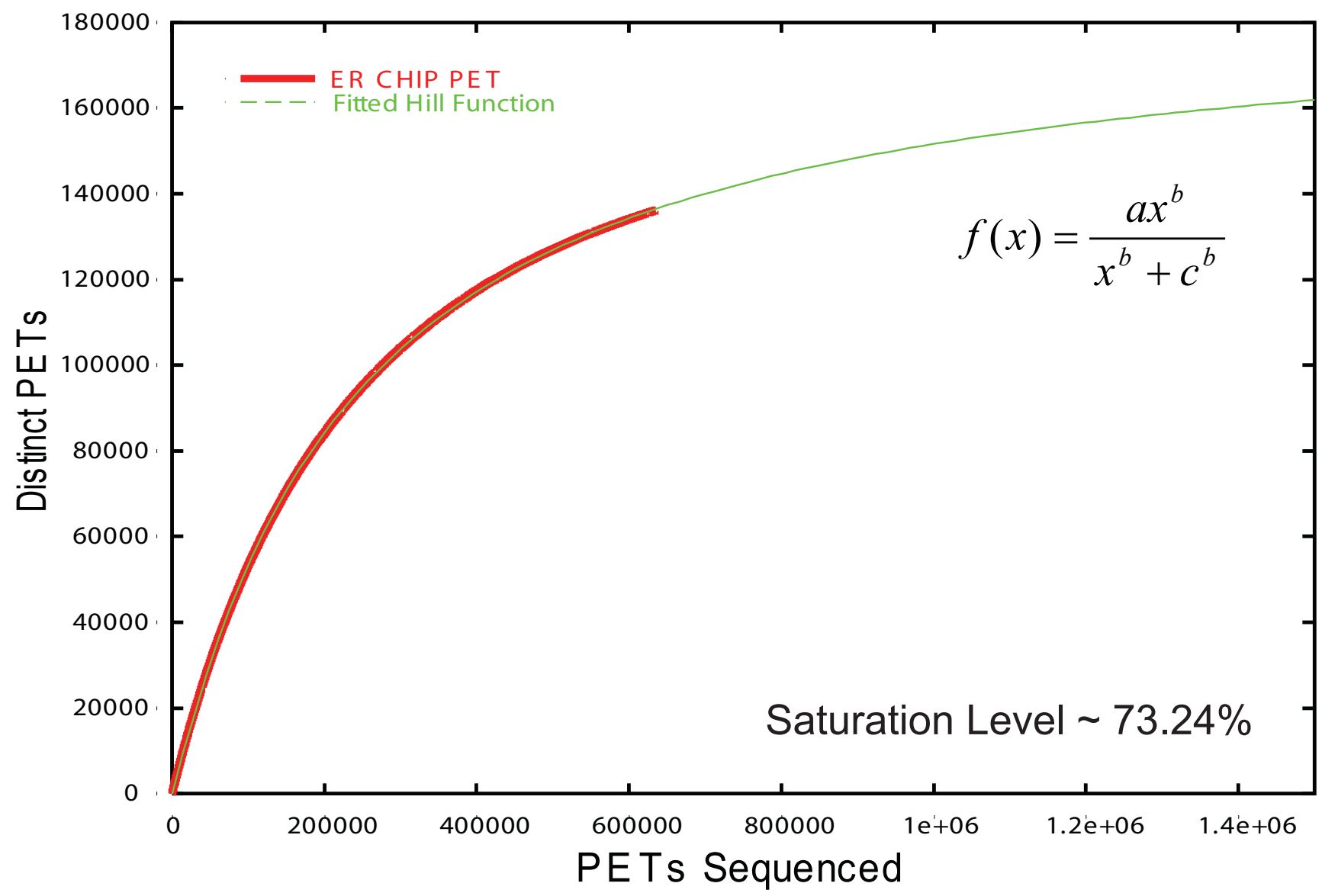

Figure S1. The number of PETs sequenced in this study represent a saturation level of 73.24%. This esimate is based on comparisons of actual sequencing results (red line) to a fitted Hill Function (green curve). The function is shown adjacent to the curves.
