# Supplementary material for: Whole-Genome Cartography of Estrogen Receptor α Binding Sites
Source: PLoS Genet. 2007 Jun 1;3(6):e87. doi: 10.1371/journal.pgen.0030087 (PMC1885282; doi:10.1371/journal.pgen.0030087)
Supplement: Supplementary file 2 [file pgen.0030087.sg002.pdf]

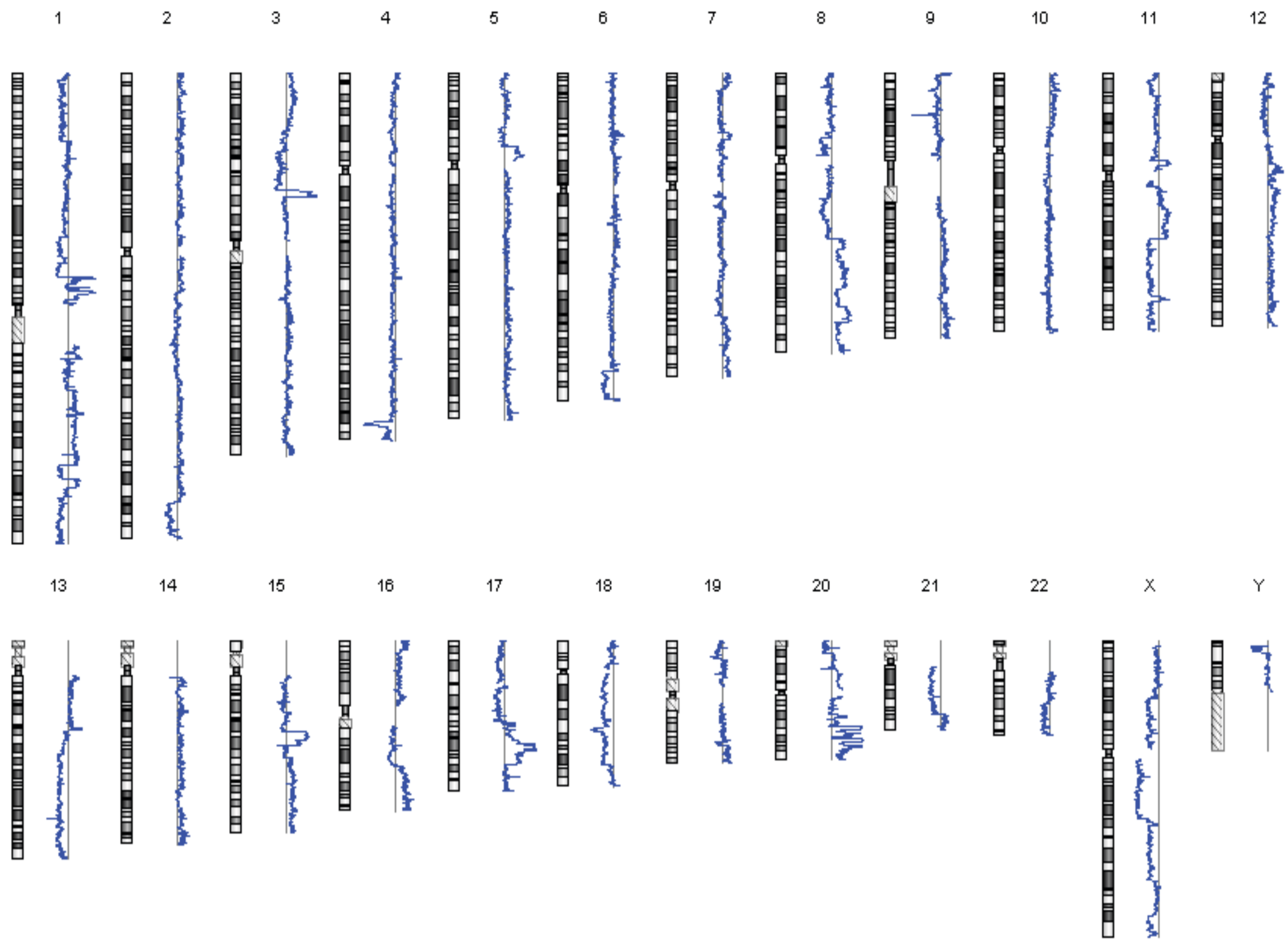

Figure S2. Comparative genome hybridization (CGH) reveals amplified regions in the MCF-7 breast cancer cell line genome.
