# Supplementary material for: Whole-Genome Cartography of Estrogen Receptor α Binding Sites
Source: PLoS Genet. 2007 Jun 1;3(6):e87. doi: 10.1371/journal.pgen.0030087 (PMC1885282; doi:10.1371/journal.pgen.0030087)
Supplement: Supplementary file 3 [file pgen.0030087.sg003.pdf]

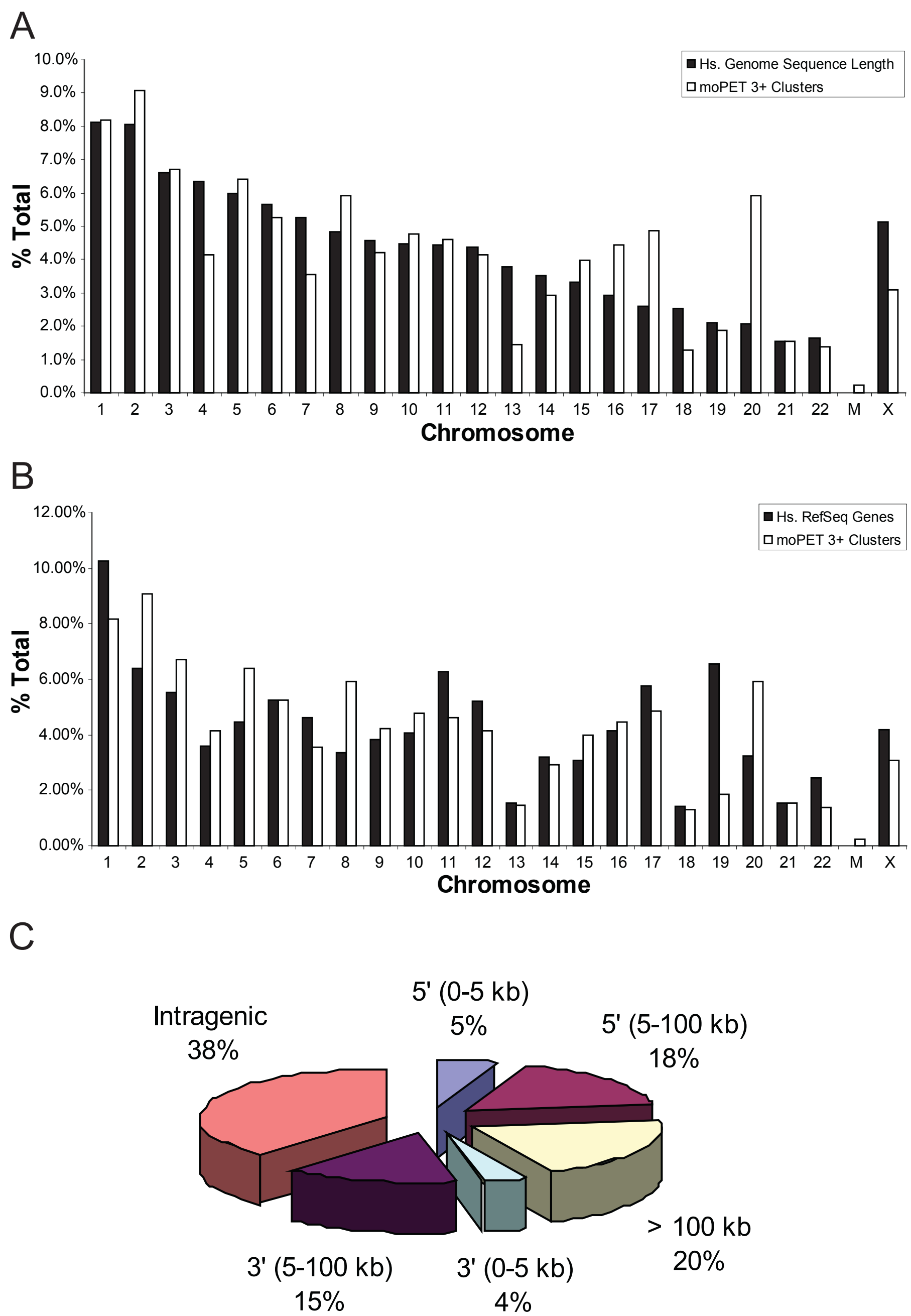

Figure S3. High confidence ER binding sites are distributed throughout the genome and are not enriched in specific chromosomes. A. Comparison of number of ChIP-PET binding sites (open bars) to chromosome size (closed bars). B. Binding site distribution (open bar) as compared to gene density (closed bars) on each chromosome. C. Locatioin of ER binding sites relative to the nearest genes in the UCSC Known Gene Database shows a large majority of sites distal to the genes (>5 kb) or within intragenic regions.
